# Debridement, antibiotics, and implant retention for periprosthetic knee infections: a pooling analysis of 1266 cases

**DOI:** 10.1186/s13018-019-1378-4

**Published:** 2019-11-12

**Authors:** Guo-Xin Qu, Cai-Hua Zhang, Shi-Gui Yan, Xun-Zi Cai

**Affiliations:** 1 Department of Orthopaedic Surgery, Second Affiliated Hospital of Zhejiang University School of Medicine, Jiefang Road 88, Hangzhou, 310009 China; 2 The affiliated Cancer Hospital of Zhengzhou University, Dongming Road 127, Zhengzhou, China; 3 Orthopaedic Research Laboratory, Zhejiang University, Jiefang Road 88, Hangzhou, China

**Keywords:** Arthroplasty, Debridement, Implant retention, Infection, Knee

## Abstract

**Background:**

The debridement, antibiotics, and implant retention (DAIR) procedure is an established therapeutic option for periprosthetic knee infections (PKI). However, the efficacy and the indication for this procedure are still controversial.

**Methods:**

All the relevant literatures were systematically reviewed and analyzed. The present study aimed to assess the success rate of DAIR in the management of PKI, identify the factors associated with prognosis of DAIR, and establish a simple algorithm for predicting a high success rate of DAIR.

**Results:**

Totally, 33 studies with 1266 cases were included. The overall success rate following DAIR in the management of PKI was 57.11%. In the subgroup analyses, the factors of “the time from symptoms to debridement was < 3 weeks” and “the bacterial species other than methicillin-resistant *Staphylococcus aureus*” significantly improved the success rate of DAIR and thus were defined as the major criteria. The statistically insignificant factors of “the open debridement and liner exchange” and “the comorbidity of rheumatoid arthritis” were set as the minor criteria. The success rate of DAIR for PKI meeting all the major criteria and no less than one minor criterion was 80.98%, which was significantly higher than the overall success rate of DAIR (*p* < 0.05).

**Conclusion:**

PKI cases meeting two major criteria and no less than one minor criterion may confer a high success rate of DAIR. This simple algorithm may contribute to identifying the appropriate PKI patient for DAIR treatment and predicting the prognosis of DAIR.

## Introduction

Periprosthetic knee infections (PKI) are disastrous complications after joint arthroplasty. The two-stage revision is the gold standard for chronic PKI [1, 2]. However, the patients have to bear two or more major surgeries, which means great sufferings and heavy economic burdens for patients [3]. Current guidelines recommend the procedure of debridement, antibiotics, and implant retention (DAIR) for the early postoperative PKI and the acute hematogenous PKI [4]. However, the success rates varied from 23 to 100% among different studies [4]. One of the plausible explanations is that the preoperative characteristics of patients, such as the timing of DAIR, the bacterial species, and the immunocompetence, are inconsistent among the studies [5, 6]. Moreover, the specific procedure of treatment, like the open debridement or the arthroscopic debridement and the liner exchange or not, may also influence the prognosis of DAIR [7]. The sensitivity of these criteria in determining outcomes is still questionable. The different preoperative characteristics of patients and the varying procedure of DAIR probably account for the inconsistent results among the literatures [5, 8].

The aims of present pooling analysis were to systematically assess the success rate of DAIR in the management of PKI, identify the factors associated with prognosis of DAIR, and establish a simple algorithm for predicting a high success rate of DAIR. To our knowledge, no such analysis has ever been made.

## Materials and methods

### Literature search

The following sources of data were searched by two reviewers: EMBASE, PubMed, Web of Science, Cochrane Central Register of Controlled Trials, and Cochrane Database of Systematic Reviews. A comprehensive search of these database was performed using the following combinations of the keywords: “knee,” “prosthesis,” “arthroplasty,” “periprosthetic,” “infection,” “debridement,” and “replacement.” No language or date restriction was applied to the search. The final search was conducted on Jan 1, 2018.

### Selection criteria

The inclusion criteria for these studies were as follows: (1) the success rate of debridement and prosthesis retaining procedure, (2) infected knee prosthesis, and (3) the data of knee DAIR which could be extracted.

The exclusion criteria were as follows: (1) prolonged suppressive antibiotic therapy after DAIR procedure, (2) sample size was < 5, (3) abstract or conference presentation without peer review, (4) the smaller series from redundant publications, and (5) two-stage revision, arthrodesis, or other therapies were included.

### Data extraction and analysis

Three authors (QGX, ZCH, and CXZ) independently screened titles and abstracts, included studies, extracted relevant data, and checked them. Any discrepancy was resolved by discussion. The following data were extracted: first author’s last name, period of the study, patient demographics, symptom duration, surgical procedure, antibiotic therapy, and outcomes.

### Assessment of trial quality

Two authors (QGX and ZCH) independently assessed the methodological quality of each study using the Newcastle-Ottawa Scale (NOS) [9]. The NOS with a maximum score of nine is validated for assessing the quality of non-randomized studies, including case-control studies and observational studies [9]. Only studies with a score of > 7 were included in the analysis. The NOS score of each study was shown in Table 1.

**Table 1:** Study characteristics, success rate of DAIR, and methodological quality based on NOS scoring system of included trials

Authors	Study period	Sample size (*n*)	Success, *n* (%)	Minimum follow-up (years)	Liner exchange	Symptom duration < 7 days, Success, *n* (%)	Symptom duration < 3 weeks, Success, *n* (%)	NOS score
Urish [10]	2005–2015	216	92 (43)	2	NR	NR	NR	7
Son [5]	2010–2014	25	22 (88)	2	yes	12 (80)	22 (88)	8
Riesgo [11]	2014–2016	36	22 (61)	1	yes	NR	22 (61)	9
Duque [12]	2006–2013	67	46 (69)	2	yes	NR	46 (69)	8
He [8]	2002–2012	11	9 (82)	2	yes	9 (82)	9 (82)	8
Nakano [13]	2000–2011	29	11 (38)	2	yes	NR	7 (47)	8
Lizauret [14]	2000–2011	39	15 (38)	3	yes	NR	NR	7
Koh [15]	2005–2012	52	37 (71)	2	part	16 (80)	37 (71)	8
Cury [6]	2008–2010	12	9 (75)	3	NR	NR	9 (75)	9
Tornero [16]	1999–2009	90	65 (72)	3	yes	NR	65 (72)	8
Chung [17]	2002–2009	16	16 (100)	2	yes	16 (100)	16 (100)	9
Stryker [18]	1995–2010	72	52 (72)	1	yes	52 (72)	52 (72)	8
Royo [19]	1996–2010	34	25 (74)	0.6	part	NR	25 (74)	9
Liu [20]	2000–2008	17	15 (88)	1	no	15 (88)	15 (88)	8
Kuiper [21]	2004–2009	29	22 (76)	2	NR	NR	22 (76)	8
Fehring [22]	1995–2009	46	17 (37)	2	part	NR	NR	8
Koyonos [23]	1996–2007	78	30 (38)	1	NR	NR	NR	8
Gardner [24]	1996–2010	44	19 (43)	1	yes	NR	19 (43)	8
Cobo [25]	2004–2016	46	29 (63)	2	yes	NR	29 (63)	9
Choi [26]	2002–2007	32	10	1	part	NR	9 (31)	8
Byrenet [27]	1998–2003	51	38 (75)	2.3	NR	NR	NR	8
Aboltins [28]	1998–2003	7	6 (86)	0.5	yes	3 (100)	5 (83)	8
Tsumura [29]	1990–2005	10	8 (80)	1	yes	7 (78)	8 (80)	8
Berdalet [30]	2000–2003	6	6 (100)	2	yes	NR	NR	8
Deirmengian [31]	1990–2000	31	11 (35)	2	NR	9 (41)	11 (41)	8
Waldman [32]	NR	16	6 (38)	2.5	no	6 (38)	6 (38)	8
Segawa [33]	1980–1995	41	28 (68)	0.3	yes	NR	27 (90)	8
Mont [34]	1984–1994	24	20 (83)	2	no	5 (100)	20 (83)	8
Wasielewski [35]	1981–1990	10	7 (70)	2	NR	NR	NR	8
Hartman [36]	1981–1989	33	13 (39)	2	NR	NR	13 (39)	8
Burger [37]	1976–1988	39	7 (18)	1	NR	4 (50)	7 (30)	8
Schoifet [38]	1973–1984	31	7 (23)	3	NR	4 (36)	5 (24)	8
Lvey [39]	1979–1988	10	5 (50)	2	NR	1 (100)	2 (40)	7

*NR* not reported, *NOS* Newcastle-Ottawa score [9]

### Pooling analysis

The pooled mean proportion of success following DAIR procedure in the management of PKI was calculated. We tried to establish a simple algorithm for predicting a high success rate of DAIR. Firstly, the factors associated with prognosis of DAIR were identified based on the included studies. Secondly, the subgroup analyses were made on those identified factors. If statistical difference was obtained, the factor was defined as the major criterion, which meant that this criterion was obligatory for the algorithm. If no statistical difference was found, the factor was defined as the minor criterion. Then, we calculated and compared the success rate of DAIR cases meeting all the major criteria and different quantity of the minor criteria. Finally, we judged how many minor criteria were needed for the algorithm.

### Statistical analysis

Information on the number of treated patients was collected. Data were calculated with the SPSS statistical software version 20.0 (SPSS Inc., Chicago, Illinois) and Microsoft Excel 2013 (Microsoft, Redmond, Washington). The data of all the outcomes conformed to the Bernoulli distribution. The weighted mean difference was calculated with 95% confidence intervals (CI) for continuous outcomes. The subgroup analysis was evaluated using a chi-squared test and Fisher’s exact test. The statistical significance was set at *p* < 0.05.

## Results

**Fig. 1:**
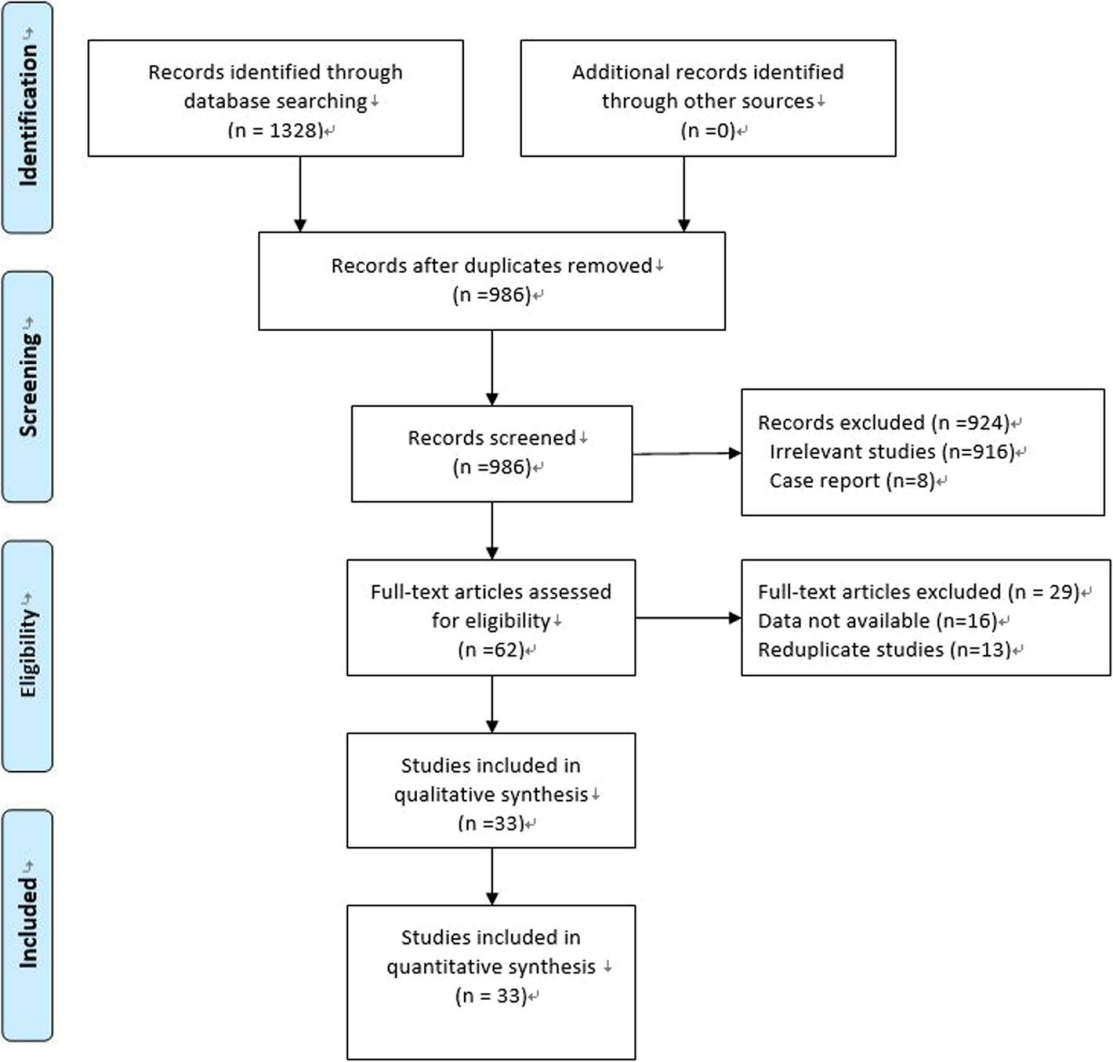
The flow diagram of this systematic review

The literature search initially yielded 1328 papers, of which 1266 papers were excluded and the full texts of the remaining 62 papers were reviewed. After careful review, 33 studies of 1266 cases were included in the present analysis (Fig. 1). The characteristics of the included studies were shown in Table 1. The mean NOS score of included studies was 8.06 (7–9). The pooled mean proportion of success following DAIR procedure in the management of PKI was 57.11% (723/1266 cases, 95% CI: 54.4–59.8%) [5, 6, 8, 10–39] (Table 1).

**Fig. 2:**
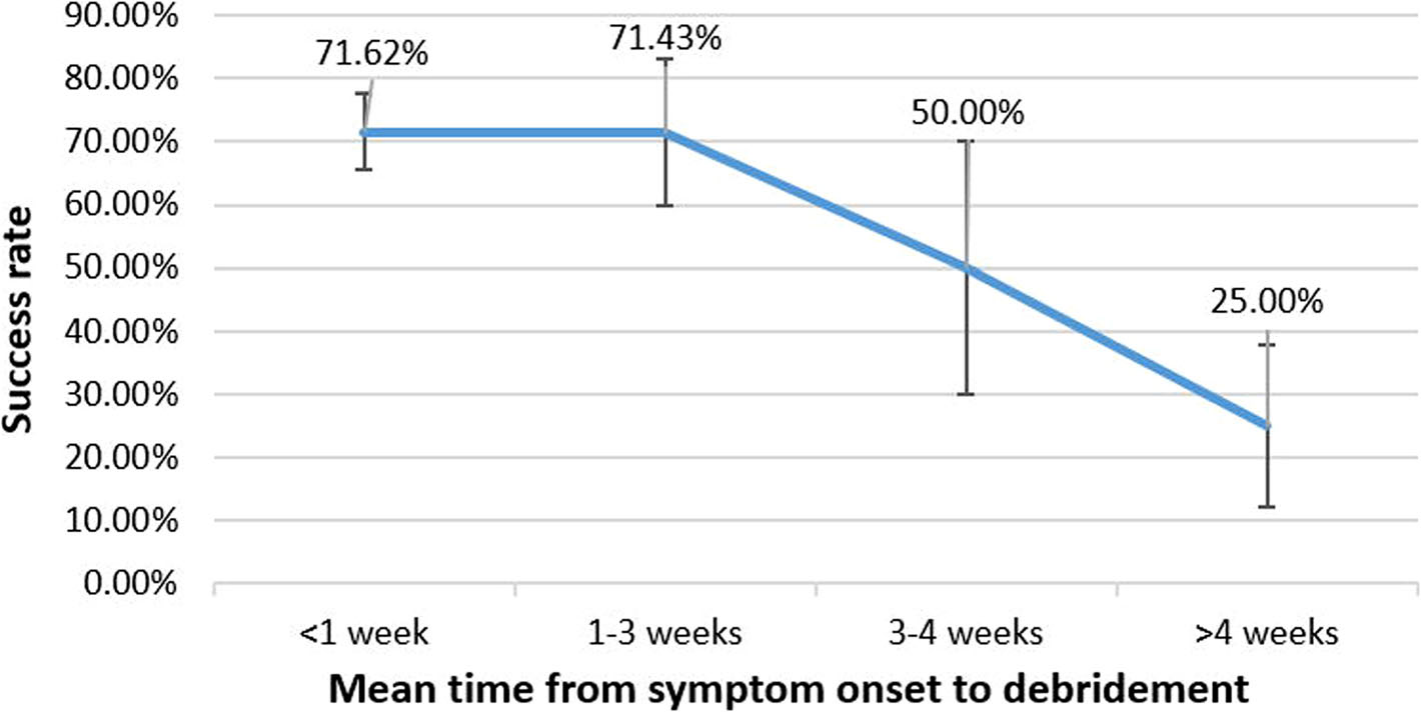
The relationship between the success rate of DAIR for treatment of PKI and the mean time from infection symptoms to debridement

The time from infection symptoms to debridement was found to be associated with the difference in the success rate of DAIR (Fig. 2). In the studies where the mean time from symptoms to debridement was < 7 days, the pooled success rate was 71.62% (159/222 cases, 95% CI: 65.6–77.6%). Similarly, in the studies where the mean time from symptoms to debridement was < 3 weeks, the pooled success rate was 71.15% (254/357 cases, 95% CI: 66–76%) [5, 15, 28, 29, 34, 37–39]. However, the pooled rate of success where the mean time from symptoms to debridement was > 3 weeks was only 35.09% (20/57 cases, 95% CI: 23–47%) [15, 32, 38, 39]. Since the difference was statistically significant (*p* < 0.05), “the time from symptoms to debridement was < 3 weeks” was set as the major criterion (Table 2).

**Table 2:** Subgroup analyses for screening the major and minor criteria of the algorithm

Factors	No. of studies	No. of cases	Infection control, % (95% CI)	RR (95% CI)	*p* value
Symptom duration					
< 3 weeks	16	357	71.15 (66–76)	0.35 (0.27–0.45)	0.000
> 3 weeks	8	57	35.09 (23–47)		
Bacterial species					
MRSA	6	40	35 (19.6–50.4)	2.06 (1.5–2.8)	0.000
Non-MRSA		168	68.5 (61.4–75.6)		
Immune situation					
RA	5	38	44.7 (29–61)	1.3 (0.93–1.83)	0.201
Non-RA		151	57.6 (50–65)		
Surgical procedure					
Open debridement and liner exchange	11	173	74 (67–81)	0.82 (0.52–1.28)	0.394
Arthroscopic debridement and liner retention	4	54	66.7 (54–79)		

*RR* relative risk, *MRSA* methicillin-resistant *Staphylococcus aureus*, *RA* rheumatoid arthritis

Six studies reported the success rates of cases with infection of methicillin-resistant *Staphylococcus aureus* (MRSA) and non-MRSA bacteria [12, 15, 17, 24, 29, 40]. The overall proportion of success for MRSA and non-MRSA bacteria was 35% (14/40 cases, 95% CI: 19.6–50.4%) and 68.5% (115/168 cases, 95% CI: 61.4–75.6%), respectively. This difference was statistically significant (*p* < 0.05). As a consequence, “the non-MRSA bacterial species” was also set as the major criterion (Table 2).

Eleven studies chose open debridement and liner exchange [17, 20, 32, 41] while four studies performed arthroscopic debridement and liner retention [17, 20, 32, 41]. The success rate was 74% (128/173 cases, 95% CI: 67–81) and 66.67% (36/54 cases, 95% CI: 54–79), respectively. The difference was not statistically significant (*p* = 0.301). Accordingly, “the open debridement and liner exchange” was set as the minor criterion (Table 2).

Five studies reported the success rates of patients with rheumatoid arthritis (RA) [5, 15, 29, 36, 38]. The success rate of RA and non-RA was 44.74% (17/38 cases, 95% CI: 29–61) and 57.6% (87/151 cases, 95% CI: 50–65), respectively. No statistical difference was found (*p* = 0.201). So “the comorbidity of RA” was also set as the minor criterion (Table 2).

After inclusion of the studies meeting two major criteria and no less than one minor criterion, the success rate of DAIR was 80.98% (149/184 cases, 95% CI: 75.3–86.7) [5, 6, 8, 12, 17, 20, 28, 29, 34, 37–39]. If the studies meeting two major criteria and two minor criteria were only included, the success rate of DAIR was 80.9% (72/89 cases, 95% CI: 73–89%) [5, 12, 29, 37]. Both the success rates were not statistically different (*p* = 0.94).

Fourteen studies were analyzed based on Tsukayama’s classification [42]. The success rate of DAIR for type II (the early infection within 30 days after the index arthroplasty) and type III (the acute hematogenous infection) was 79.17% (95/120 cases, 95% CI: 71.8–86.5) and 61.65% (164/266 cases, 95% CI: 55.8–67.5), respectively. The difference was statistically different (*p* < 0.05) [5, 8, 15, 17, 18, 20, 24, 26, 28, 29, 32–34, 36] (Table 3).

**Table 3:** The difference in success rate of DAIR between early postoperative infections and acute hematogenous infections

Authors	Total success, *n* (%)	Early postoperative infections, Success, *n* (%)	Acute hematogenous infections, Success, *n* (%)
Son [5]	22 (88)	10 (100)	12 (80)
He [8]	9 (82)	0 (0)	9 (82)
Koh [15]	37 (71)	26 (81)	11 (55)
Chung [17]	16 (100)	4 (100)	12 (100)
Stryker [18]	52 (72)	0 (0)	52 (72)
Liu [20]	15 (88)	0 (0)	15 (88)
Gardner [24]	19 (43)	5 (50)	14 (41)
Choi [26]	10 (31)	2 (33)	7 (30)
Aboltins [28]	6 (86)	4 (80)	2 (100)
Tsumura [29]	8 (100)	3 (60)	5 (100)
Waldman [32]	6 (38)	2 (50)	4 (33)
Segawa [33]	28 (68)	22 (96)	5 (71)
Mont [34]	20 (83)	10 (100)	10 (71)
Hartman [36]	13 (39)	7 (64)	6 (27)

Multiple debridements were chosen in ten studies if a single debridement fail [14, 17, 18, 20, 28, 29, 32–34, 43]. The success rate of a single debridement and multiple debridements was 57.23% (190/332 cases, 95% CI: 51.9–62.6) and 49.25% (33/67 cases, 95% CI: 37.0–61.5), respectively. The difference was not statistically different (*p* = 0.281).

## Discussion

There are controversies about the therapeutic efficacy of DAIR on PKI. This pooling analysis is the first to establish a simple algorithm for predicting a high success rate of DAIR in treatment of PKI. The most important finding was that the PKI cases meeting two major criteria (“the time from symptoms to debridement was < 3 weeks” and “the non-MRSA bacterial species”), and no less than one minor criterion (“the open debridement and liner exchange” and “the comorbidity of RA”) conferred a success rate of 80.98% of DAIR. Additionally, the success rate of DAIR is significantly higher for the Tsukayama’s type II infection compared with the type III infection.

Although it is agreed that DAIR should be carried out as soon as possible for fear of biofilm formation, the deadline of DAIR is controversial. Hartman et al. [36] performed DAIR within 4 weeks from symptom onset to debridement. Cury et al. [6] started DAIR within 3 weeks after onset of infection symptoms. In a previous meta-analysis, Tsang et al. [44] reported a similar success rate of 64.7% in infected hip arthroplasty treated with DAIR. They found that the success rate was significantly higher when the time from symptoms to debridement was < 7 days compared with > 7 days. In clinical practice, however, it is difficult to perform DAIR timely within 7 days after symptoms of PKI. In the present analysis, we showed that there was no significant difference in the success rate of DAIR between < 7 days and < 3 weeks. The success rate sharply dropped after 3 weeks. Resultingly, we suggest that the time limit of DAIR for PKI could be prolonged to 3 weeks. “The time from symptoms to debridement was < 3 weeks” was set as the major criterion of the algorithm for predicting a high success rate of DAIR. It is assumed that the difference in infection site may partly account for the difference of time limit between Tsang’s analysis and the present study.

It is well accepted that the infection of MRSA is associated with a high failure rate of DAIR. The study by Bradbury et al. [40] showed that the success rate of DAIR against antibiotic-resistance bacteria including MRSA was strikingly lower than that against antibiotic-sensitive bacteria. Even in antibiotic-resistance bacteria, Peel et al. [45] found that MRSA was more likely to fail treatment than methicillin-resistant coagulase-negative staphylococci. Duque et al. [12] reported only 20% of the success rate of DAIR for MRSA infections. Our present analysis demonstrated that MRSA had a relative risk of treatment failure of 2.06 compared with non-MRSA bacteria, which agrees with the previous literatures. Since “non-MRSA infection” is an independent predicting factor for success of DAIR treatment, it was chosen as the major criterion.

Previous studies showed that open debridement and liner exchange could reduce bacterial load and increase the success rate of DAIR [7, 44]. Furthermore, it is reported that liner exchange was a strong predictor for treatment success of DAIR [46]. In the present pooling analysis, the success rate of DAIR with open debridement and liner exchange was slightly higher than that with arthroscopic debridement and liner retention though the difference was statistically insignificant. We note that the times from symptoms to debridement were both less than 1 week for two groups, which may bias the comparison. Thus, “open debridement and liner exchange” was set as the minor criterion of DAIR success.

It is believed that the patients with poor immunocompetence, such as RA, had an inferior resistance to PKI. Berbari et al. [47] reported only 32% of success rate of DAIR for prosthetic joint infections in patients with RA. In the present study, the patients with RA presented a lower success rate of DAIR compared with those without RA. Considering no statistical significance for the factor of RA, “the comorbidity of RA” was set as the minor criterion of DAIR success.

The present analysis conferred a low success proportion of 57.11% for PKI treated with DAIR, which is similar to what was reported in previous meta-analyses. Romano et al. [48] reported a low success rate of 45.9% after DAIR in hip and knee prosthesis infections. Tsang et al. [44] showed that the success rate following DAIR in the management of an infected hip arthroplasty was 64.7%. We aimed to establish a practical and simple algorithm as a tool for improving the success rate of DAIR. After selecting patients who meet two major criteria and no less than one minor criterion, the success rate of DAIR increased to over 80%. The algorithm may provide a reference of choosing the appropriate patient for DAIR treatment.

Our subgroup analysis showed that the early postoperative infection presented a higher success rate of DAIR than the acute hematogenous infection, which accords with the previous reports [15, 49]. Two reasons possibly account for it: (1) the actual duration of symptoms in the acute hematogenous infection is always longer than what is recalled by the patient, and (2) some acute hematogenous infection was essentially the acute exacerbation of a latent chronic infection.

There were limitations in our study. Firstly, the results were limited by the relative low quality and the heterogeneity of the available data. Ignoring this source of data would have underpowered the analysis and influenced the accuracy of our findings. Therefore, the present data have to be interpreted with caution, given the biases. Secondly, due to the relatively small sample size in subgroup analyses, further robust researches like randomized controlled trials and case-control trials will be indispensable for confirming the criteria and algorithm.

## Conclusion

The overall success proportion of DAIR for treating PKI is 57.11%. In our established algorithm, PKI cases meeting two major criteria (“the time from symptoms to debridement was < 3 weeks” and “the non-MRSA bacterial species”) and no less than one minor criterion (“the open debridement and liner exchange” and “the comorbidity of RA”) conferred a success rate of 80.98% of DAIR. This simple algorithm may contribute to identifying the appropriate patient for DAIR treatment and predicting the prognosis of DAIR in clinical practice.

## Data Availability

The data of the manuscript was presented in the paper and additional files.

## Abbreviation

DAIR: Antibiotics and implant retention
MRSA: Methicillin-resistant *Staphylococcus aureus*
PKI: Periprosthetic knee infections
RA: Rheumatoid arthritis
